# *Z*-Distance Based IF-THEN Rules

**DOI:** 10.1155/2016/1673537

**Published:** 2016-04-27

**Authors:** R. A. Aliev, O. H. Huseynov, R. X. Zulfugarova

**Affiliations:** ^1^Joint MBA Program, USA, Azerbaijan, Azerbaijan State Oil and Industry University, 20 Azadlig Avenue, 1010 Baku, Azerbaijan; ^2^Department of Computer Engineering, Near East University, Lefkoşa, Northern Cyprus, Mersin 10, Turkey; ^3^Department of Computer Engineering, Azerbaijan State Oil and Industry University, 20 Azadlig Avenue, 1010 Baku, Azerbaijan

## Abstract

Decision making, reasoning, and analysis in real-world problems are complicated by imperfect information. Real-world imperfect information is mainly characterized by two features. In view of this, Professor Zadeh suggested the concept of a *Z*-number as an ordered pair *Z* = (*A*, *B*) of fuzzy numbers *A* and *B*, the first of which is a linguistic value of a variable of interest, and the second one is a linguistic value of probability measure of the first one, playing a role of its reliability. The concept of distance is one of the important concepts for handling imperfect information in decision making and reasoning. In this paper, we, for the first time, apply the concept of distance of *Z*-numbers to the approximate reasoning with *Z*-number based IF-THEN rules. We provide an example on solving problem related to psychological issues naturally characterized by imperfect information, which shows applicability and validity of the suggested approach.

## 1. Introduction

Decision making, reasoning, and analysis in real-world problems are complicated by imperfect information. Real-world imperfect information is mainly characterized by two features. On the one hand, real-world information is often described on a basis of perception, experience, and knowledge of a human being. In turn, these operate with linguistic description carrying imprecision and vagueness, for which fuzzy sets based formalization can be used. On the other side, perception, experience, and knowledge of a human being are not sources of the truth. Therefore, the reliability is a degree of a partial confidence of a human being, which is naturally partial. This partial reliability is also naturally imprecise and can be formalized as a fuzzy value of probability measure. In order to ground the formal basis for dealing with real-world information, Zadeh suggested the concept of a *Z*-number [1] as an ordered pair *Z* = (*A*, *B*) of continuous fuzzy numbers used to describe a value of a random variable *X*, where *A* is a fuzzy constraint on values of *X* and *B* is a fuzzy reliability of *A* and is considered as a value of probability measure of *A*. Nowadays a series of works devoted to *Z*-numbers and their application in decision making, control, and other fields [2–13] exists. A general and computationally effective approach to computation with discrete *Z*-numbers is suggested in [14–16]. The authors provide motivation of the use of discrete *Z*-numbers mainly based on the fact that NL-based information is of a discrete framework. The suggested arithmetic of discrete *Z*-numbers includes basic arithmetic operations and important algebraic operations.

The concept of distance is one of the important concepts for decision making and reasoning [17, 18]. In this paper, we for the first time apply the concept of distance of *Z*-numbers to the approximate reasoning with *Z*-number based IF-THEN rules. An approximate reasoning refers to a process of inferring imprecise conclusions from imprecise premises [17–38]. As one can see, this process often takes place in various fields of human activity including economics, decision analysis, system analysis, control, and everyday activity. The reason for this is that information relevant to real-world problems is, as a rule, imperfect. According to Zadeh, imperfect information is information which in one or more respects is imprecise, uncertain, incomplete, unreliable, vague, or partially true [39]. We can say that in a wide sense approximate reasoning is reasoning with imperfect information.

The paper is structured as follows. In Section 2, we present some prerequisite material including definitions of a discrete fuzzy number, a discrete *Z*-number, and probability measure of a discrete fuzzy number. In Section 3, we propose several distance measures for *Z*-numbers. In Section 4, we describe the statement of the problem and the suggested approach to reasoning with *Z*-rules on the basis of distance of *Z*-numbers. In Section 5, we illustrate an application of the suggested approach to a real-world problem which involves modeling of psychological aspects.  Section 6 concludes.

## 2. Preliminaries

### 2.1. Main Definitions

**Definition 1 (a discrete fuzzy number [40–43]).**

A fuzzy subset *A* of the real line *ℛ* with membership function *μ*_*A*_ : *ℛ* → [0,1] is a discrete fuzzy number if its support is finite; that is, there exist *x*_1_,…, *x*_*n*_ ∈ *ℛ* with *x*_1_ < *x*_2_ < ⋯<*x*_*n*_, such that supp⁡(*A*) = {*x*_1_,…, *x*_*n*_} and there exist natural numbers *s*, *t* with 1 ≤ *s* ≤ *t* ≤ *n* satisfying the following conditions:

(1) *μ*_*A*_(*x*_*i*_) = 1 for any natural number *i* with *s* ≤ *i* ≤ *t*;
(2) *μ*_*A*_(*x*_*i*_) ≤ *μ*_*A*_(*x*_*j*_) for natural numbers *i*, *j* with 1 ≤ *i* ≤ *j* ≤ *s*;
(3) *μ*_*A*_(*x*_*i*_) ≥ *μ*_*A*_(*x*_*j*_) for natural numbers *i*, *j* with *t* ≤ *i* ≤ *j* ≤ *n*.

**Definition 2 (a discrete random variable and a discrete probability distribution [44]).**

A random variable, *X*, is a variable whose possible values *x* are outcomes of a random phenomenon. A discrete random variable is a random variable which takes only a countable set of its values *x*.

Consider a discrete random variable *X* with outcomes space {*x*_1_,…, *x*_*n*_}. A probability of an outcome *X* = *x*_*i*_, denoted *P*(*X* = *x*_*i*_), is defined in terms of a probability distribution. A function *p* is called a discrete probability distribution or a probability mass function if

(1) $$\begin{array}{c} P\left(\;X=x_{i}\;\right)=p\left(\;x_{i}\;\right), \end{array}$$

where *p*(*x*_*i*_)∈[0,1] and ∑_*i*=1_^*n*^ *p*(*x*_*i*_) = 1.

**Definition 3 (arithmetic operations over discrete random variables [44, 45]).**

Let *X*_1_ and *X*_2_ be two independent discrete random variables with the corresponding outcome spaces *X*_1_ = {*x*_11_,…, *x*_1*i*_,…, *x*_1*n*_1__} and *X*_2_ = {*x*_21_,…, *x*_2*i*_,…, *x*_2*n*_2__} and the corresponding discrete probability distributions *p*_1_ and *p*_2_. The probability distribution of *X*_12_ = *X*_1_ *∗X*_2_, *∗* ∈ {+, −, ·, /}, is the convolution *p*_12_ = *p*_1_∘*p*_2_ of *p*_1_ and *p*_2_ which is defined for any *x* ∈ {*x*_1_ *∗x*_2_∣*x*_1_ ∈ *X*_1_, *x*_2_ ∈ *X*_2_}, *x*_1_ ∈ *X*_1_, *x*_2_ ∈ *X*_2_, as follows:

(2) $$\begin{array}{c} p_{12}\left(\;x\;\right)=\underset{x=x_{1}*x_{2}}{\sum }p_{1}\left(\;x_{1}\;\right)p_{2}\left(\;x_{2}\;\right). \end{array}$$

**Definition 4 (probability measure of a discrete fuzzy number [46]).**

Let *X* be discrete random variable with probability distribution *p*. Let *A* be a discrete fuzzy number describing a possibilistic restriction on values of *X*. A probability measure of *A* denoting *P*(*A*) is defined as

(3) PA∑i=1nμAxipxi=μAx1px1+μAx2px2+⋯+μAxnpxn.

**Definition 5 (a scalar multiplication of a discrete fuzzy number [16]).**

A scalar multiplication of a discrete fuzzy number *A* by a real number *λ* ∈ *ℛ* is the discrete fuzzy number *A*_1_ = *λA*, whose *α*-cut is defined as

(4) A1α=x∈λ·supp⁡A ∣ min⁡λAα≤x≤max⁡λAα,

where

(5) λ·supp⁡A=λx ∣ x∈supp⁡A,min⁡λAα=min⁡λx ∣ x∈Aα,max⁡λAα=max⁡λx ∣ x∈Aα,

and the membership function is defined as

(6) $$\begin{array}{c} \mu_{\lambda A}\left(\;x\;\right)=\sup\left\{\;\alpha \in \left[\;0,1\;\right]\;|\;x\in \left(\;\lambda A^{\alpha }\;\right)\;\right\}. \end{array}$$

**Definition 6 (addition of discrete fuzzy numbers [40–43]).**

For discrete fuzzy numbers *A*_1_, *A*_2_, their addition *A*_12_ = *A*_1_ + *A*_2_ is the discrete fuzzy number whose *α*-cut is defined as

(7) A12α=x∈supp⁡A1+supp⁡A2 ∣ min⁡A1α+A2α≤x≤max⁡A1α+A2α,

where supp⁡(*A*_1_) + supp⁡(*A*_2_) = {*x*_1_ + *x*_2_∣*x*_*j*_ ∈ supp⁡(*A*_*j*_), *j* = 1,2}, min⁡{*A*_1_^*α*^ + *A*_2_^*α*^} = min{*x*_1_ + *x*_2_∣*x*_*j*_ ∈ *A*_*j*_^*α*^,  *j* = 1,2}, max⁡{*A*_1_^*α*^ + *A*_2_^*α*^} = max⁡{*x*_1_ + *x*_2_∣*x*_*j*_ ∈ *A*_*j*_^*α*^, *j* = 1,2}, and the membership function is defined as

(8) $$\begin{array}{c} \mu_{A_{1}+A_{2}}\left(\;x\;\right)=\sup\left\{\;\alpha \in \left[\;0,1\;\right]\;|\;x\in \left\{\;A_{1}^{\alpha }+A_{2}^{\alpha }\;\right\}\;\right\}. \end{array}$$

**Definition 7 (a discrete *Z*-number [15, 16]).**

A discrete *Z*-number is an ordered pair *Z* = (*A*, *B*) of discrete fuzzy numbers *A* and *B*. *A* plays a role of a fuzzy constraint on values that a random variable *X* may take. *B* is a discrete fuzzy number with a membership function *μ*_*B*_ : {*b*_1_,…, *b*_*n*_}→[0,1], {*b*_1_,…, *b*_*n*_}⊂[0,1], playing a role of a fuzzy constraint on the probability measure of *A*, *P*(*A*) = ∑_*i*=1_^*n*^ *μ*_*A*_(*x*_*i*_)*p*(*x*_*i*_), *P*(*A*) ∈ supp⁡(*B*).

## 3. Distance between Two *Z*-Numbers

Denote by *ℱ* the space of discrete fuzzy sets of *ℛ*. Denote by *ℱ*_[*a*,*b*]_ the space of discrete fuzzy sets of [*a*, *b*] ⊂ *ℛ*.

**Definition 8 (the supremum metric on *𝒟* [47]).**

The supremum metric *d* on *ℱ* is defined as

(9) dA1,A2=sup⁡dHA1α,A2α ∣ 0<α≤1,A1,A2∈F,

where *d*_*H*_ is the Hausdorff distance.

(*ℱ*, *d*) is a complete metric space [47, 48].

**Definition 9 (fuzzy Hausdorff distance [16]).**

The fuzzy Hausdorff distance *d*_*fH*_ between *A*_1_, *A*_2_ ∈ *ℱ* is defined as

(10) $$\begin{array}{c} d_{fH}\left(\;A_{1},A_{2}\;\right)=\underset{\alpha \in \left[0,1\right]}{⋃}\alpha d_{fH}^{\alpha }\left(\;A_{1},A_{2}\;\right), \end{array}$$

where

(11) $$\begin{array}{c} d_{fH}^{\alpha }\left(\;A_{1},A_{2}\;\right)=\left.\;\left\{\;\underset{\alpha \leq \overset{-}{\alpha }\leq 1}{\sup}d_{H}\left(\;A_{1}^{\overset{-}{\alpha }},A_{2}^{\overset{-}{\alpha }}\;\right)\;\right.\;\right\}, \end{array}$$

where $\overset{-}{\alpha }$ is the value which is within *α*-cut and 1-cut. (*ℱ*, *d*_*fH*_) is a complete metric space.

Denote by *𝒵* the space of discrete *Z*-numbers:

(12) $$\begin{array}{c} Z=\left\{\;Z=\left(\;A,B\;\right)\;|\;A\in F,\;\;B\in F_{\left[0,1\right]}^{}\;\right\}. \end{array}$$

**Definition 10 (supremum metrics on *𝒵* [16]).**

The supremum metrics on *𝒵* are defined as

(13) $$\begin{array}{c} D\left(\;Z_{1},Z_{2}\;\right)=d\left(\;A_{1},A_{2}\;\right)+d\left(\;B_{1},B_{2}\;\right); \end{array}$$

(*𝒵*, *D*) is a complete metric space. This follows from the fact that (*ℱ*, *d*) is a complete metric space.

*D*(*Z*_1_, *Z*_2_) has the following properties:

(14) DZ1+Z,Z2+Z=DZ1,Z2,DZ2,Z1=DZ1,Z2,DλZ1,λZ2=λDZ1,Z2,λ∈R,DZ1,Z2≤DZ1,Z+DZ,Z2.

**Definition 11 (fuzzy Hausdorff distance between *Z*-numbers [16]).**

The fuzzy Hausdorff distance *d*_*fHZ*_ between *Z*-numbers *Z*_1_ = (*A*_1_, *B*_1_), *Z*_2_ = (*A*_2_, *B*_2_) ∈ *𝒵* is defined as

(15) $$\begin{array}{c} d_{fHZ}\left(\;Z_{1},Z_{2}\;\right)=d_{fH}\left(\;A_{1},A_{2}\;\right)+d_{fH}\left(\;B_{1},B_{2}\;\right). \end{array}$$

**Definition 12 (*Z*-valued Euclidean distance between discrete *Z*-numbers [16]).**

Given two discrete *Z*-numbers *Z*_1_ = (*A*_1_, *B*_1_), *Z*_2_ = (*A*_2_, *B*_2_) ∈ *𝒵*, *Z*-valued Euclidean distance *d*_*E*_(*Z*_1_, *Z*_2_) between *Z*_1_ and *Z*_2_ is defined as

(16) $$\begin{array}{c} d_{E}\left(\;Z_{1},Z_{2}\;\right)=\sqrt{{\left(Z_{1}-Z_{2}\right)}^{2}}. \end{array}$$

## 4. *Z*-Valued IF-THEN Rules Based Reasoning

A problem of interpolation of *Z*-rules termed as *Z*-interpolation was addressed by Zadeh as a challenging problem [33]. This problem is the generalization of interpolation of fuzzy rules [49]. The problem of *Z*-interpolation is given below.

Given the following *Z*-rules,

 if *X*_1_ is *Z*_*X*_1_,1_ = (*A*_*X*_1_,1_, *B*_*X*_1_,1_) and so on and *X*_*m*_ is *Z*_*X*_*m*_,1_ = (*A*_*X*_*m*_,1_, *B*_*X*_*m*_,1_), then *Y* is *Z*_*Y*_ = (*A*_*Y*,1_, *B*_*Y*,1_),
 if *X*_1_ is *Z*_*X*_1_,2_ = (*A*_*X*_1_,2_, *B*_*X*_1_,2_) and so on and *X*_*m*_ is *Z*_*X*_*m*_,2_ = (*A*_*X*_*m*_,2_, *B*_*X*_*m*_,2_), then *Y* is *Z*_*Y*_ = (*A*_*Y*,2_, *B*_*Y*,2_),
 if *X*_1_ is *Z*_*X*_1_,*n*_ = (*A*_*X*_1_,*n*_, *B*_*X*_1_,*n*_) and so on and *X*_*m*_ is *Z*_*X*_*m*_,*n*_ = (*A*_*X*_*m*_,*n*_, *B*_*X*_*m*_,*n*_) then *Y* is *Z*_*Y*_ = (*A*_*Y*,*n*_, *B*_*Y*,*n*_),

and a current observation

 
*X*_1_ is *Z*_*X*_1__′ = (*A*_*X*_1__′, *B*_*X*_1__′) and so on and *X*_*m*_ is *Z*_*X*_*m*__′ = (*A*_*X*_*m*__′, *B*_*X*_*m*__′),

find the *Z*-value of *Y*. Here *m* is the number of *Z*-valued input variables and *n* is the number of rules.

The idea underlying the suggested interpolation approach is that the ratio of distances between the resulting output and the consequent parts is equal to one between the current input and the antecedent parts [49]. This implies for *Z*-rules that the resulting output *Z*_*Y*_′ is computed as

(17) $$\begin{array}{c} Z_{Y}^{\prime }=\overset{n}{\underset{j=1}{\sum }}w_{j}Z_{Y,j}=\overset{n}{\underset{j=1}{\sum }}w_{j}\left(\;A_{Y,j},B_{Y,j}\;\right), \end{array}$$

where *Z*_*Y*,*j*_ is the *Z*-number valued consequent of the *j*th rule, *w*_*j*_ = (1/*ρ*_*j*_)/(∑_*j*=1_^*n*^1/*ρ*_*j*_), *j* = 1,…, *n* are coefficients of linear interpolation, and *n* is the number of *Z*-rules. *ρ*_*j*_ = ∑_*i*=1_^*m*^ *D*(*Z*_*X*_*i*__′, *Z*_*X*_*i*_,*j*_), where *D* is the distance between current *i*th *Z*-number valued input and the *i*th *Z*-number valued antecedent of the *j*th rule. Thus, *ρ*_*j*_ computes the distance between a current input vector and the vector of the antecedents of *j*th rule.

In this paper, we will consider discrete *Z*-numbers. The operations of addition and scalar multiplication of discrete *Z*-numbers are described below.

*Addition of Discrete Z-Numbers*. Let *Z*_1_ = (*A*_1_, *B*_1_) and *Z*_2_ = (*A*_2_, *B*_2_) be discrete *Z*-numbers describing imperfect information about values of variables *X*_1_ and *X*_2_. Consider the problem of computation of addition *Z*_12_ = *Z*_1_ + *Z*_2_. The first stage is the computation addition of discrete fuzzy numbers *A*_12_ = *A*_1_ + *A*_2_ on the basis of Definition 6.

The second stage involves stage-by-stage construction of *B*_12_ which is related to propagation of probabilistic restrictions. We realize that, in *Z*-numbers *Z*_1_ = (*A*_1_, *B*_1_) and *Z*_2_ = (*A*_2_, *B*_2_), the “true” probability distributions *p*_1_ and *p*_2_ are not exactly known. In contrast, the information available is represented by the fuzzy restrictions:

(18) $$\begin{array}{c} \overset{n_{1}}{\underset{k=1}{\sum }}\mu_{A_{1}}\left(\;x_{1k}\;\right)p_{1}\left(\;x_{1k}\;\right)\text{ is }B_{1},\\ \overset{n_{2}}{\underset{k=1}{\sum }}\mu_{A_{2}}\left(\;x_{2k}\;\right)p_{2}\left(\;x_{2k}\;\right)\text{ is }B_{2}, \end{array}$$

which are represented in terms of the membership functions as

(19) $$\begin{array}{c} \mu_{B_{1}}\left(\;\overset{n_{1}}{\underset{k=1}{\sum }}\mu_{A_{1}}\left(\;x_{1k}\;\right)p_{1}\left(\;x_{1k}\;\right)\;\right),\\ \mu_{B_{2}}\left(\;\overset{n_{2}}{\underset{k=1}{\sum }}\mu_{A_{2}}\left(\;x_{2k}\;\right)p_{2}\left(\;x_{2k}\;\right)\;\right). \end{array}$$

Thus, one has the fuzzy sets of probability distributions of *p*_1_ and *p*_2_ with the membership functions defined as

(20) μp1p1=μB1∑k=1n1μA1x1kp1x1k,μp2p2=μB2∑k=1n2μA2x2kp2x2k.

Therefore, we should construct these fuzzy sets. *B*_*j*_, *j* = 1,2, is a discrete fuzzy number which plays the role of a soft constraint on a value of a probability measure of *A*_*j*_. Therefore, basic values *b*_*jl*_ ∈ supp⁡(*B*_*j*_), *j* = 1,2, *l* = 1,…, *m*, of a discrete fuzzy number *B*_*j*_, *j* = 1,2, are values of a probability measure of *A*_*j*_, *b*_*jl*_ = *P*(*A*_*j*_). Thus, given *b*_*jl*_, we have to find such probability distribution *p*_*jl*_ which satisfies

(21) bjl=μAjxj1pjlxj1+μAjxj2pjlxj2+⋯+μAjxjnjpjlxjnj.

At the same time, we know that *p*_*jl*_ has to satisfy

(22) $$\begin{array}{c} \overset{n_{j}}{\underset{k=1}{\sum }}p_{jl}\left(\;x_{jk}\;\right)=1,\;p_{jl}\left(x_{jk}\right)\geq 0. \end{array}$$

Thus, the following goal programming problem should be solved to find *p*_*j*_:

(23) μAjxj1pjlxj1+μAjxj2pjlxj2+⋯+μAjxjnjpjlxjnj⟶bjl,

subject to

(24) pjlxj1+pjlxj2+⋯+pjlxjnj=1,pjlxj1,pjlxj2,…,pjlxjnj≥0.

For each *l* = 1,…, *m* and each *k* = 1,…, *n*_*j*_ denote *c*_*k*_ = *μ*_*A*_*j*__(*x*_*jk*_) and *v*_*k*_^*l*^ = *p*_*jl*_(*x*_*jk*_), *k* = 1,…, *n*_*j*_. As *c*_*k*_ and *b*_*jl*_ are known and *v*_*k*_^*l*^ are unknown, we see that problem (23)-(24) is nothing but the following goal linear programming problem:

$$\begin{array}{cc} \left(23^{\prime }\right) & c_{1}v_{1}^{l}+c_{2}v_{2}^{l}+\cdots +c_{n}v_{n}^{l}⟶b_{jl}, \end{array}$$

subject to

24′v1l+v2l+⋯+vnl=1,v1l,v2l,…,vnl≥0.

Having obtained the solution *v*_*k*_^*l*^, *k* = 1,…, *n*_*j*_, of problems 23′-24′ for each *l* = 1,…, *m*, recall that *v*_*k*_^*l*^ = *p*_*jl*_(*x*_*jk*_), *k* = 1,…, *n*_*j*_. As a result, *p*_*jl*_(*x*_*jk*_), *k* = 1,…, *n*_*j*_, is found, and, therefore, distribution *p*_*jl*_ is obtained. Thus, to construct *μ*_*p*_*jl*__, we need to solve *m* simple problems 23′-24′. Let us mention that in general, problems 23′-24′ do not have a unique solution. In order to guarantee existence of a unique solution, the compatibility conditions can be included:

(25) $$\begin{array}{c} \overset{n_{j}}{\underset{k=1}{\sum }}x_{jk}p_{jl}\left(\;x_{jk}\;\right)=\frac{\sum_{k=1}^{n_{j}}x_{jk}\mu_{A_{j}}\left(x_{jk}\right)}{\sum_{k=1}^{n_{j}}\mu_{A_{j}}\left(x_{jk}\right)}. \end{array}$$

This condition implies that the centroid of *A*_*j*_ is to coincide with that of *p*_*jl*_.

Probability distributions *p*_*jl*_(*x*_*jk*_), *k* = 1,…, *n*_*j*_, naturally induce probabilistic uncertainty over the result *X* = *X*_1_ + *X*_2_. This implies, given any possible pair *p*_1*l*_, *p*_2*l*_ of the extracted distributions, the convolution *p*_12*s*_ = *p*_1*l*_∘*p*_2*l*_, *s* = 1,…, *m* ^2^, is to be computed as follows:

(26) p12x=∑x1+x2=xp1lx1p2lx2,∀x∈X12;  x1∈X1,  x2∈X2.

Given *p*_12*s*_, the value of probability measure of *A*_12_ can be computed:

(27) $$\begin{array}{c} P\left(\;A_{12}\;\right)=\overset{n}{\underset{k=1}{\sum }}\mu_{A_{12}}\left(\;x_{12k}\;\right)p_{12}\left(\;x_{12k}\;\right). \end{array}$$

However, the “true” *p*_12*s*_ is not exactly known as the “true” *p*_1*l*_ and *p*_2*l*_ are described by fuzzy restrictions. In other words, the fuzzy sets of probability distributions *p*_1*l*_ and *p*_2*l*_ induce the fuzzy set of convolutions *p*_12*s*_, *s* = 1,…, *m* ^2^, with the membership function defined as

(28) $$\begin{array}{c} \mu_{p_{12}}\left(\;p_{12}\;\right)=\underset{p_{1},p_{2}}{\max}\left[\;\mu_{p_{1}}\left(\;p_{1}\;\right)\wedge \mu_{p_{2}}\left(\;p_{2}\;\right)\;\right], \end{array}$$

subject to

(29) p12=p1∘p2,μpjpj=μBj∑k=1njμAjxjkpjlxjk,

where ∧ is min operation.

As a result, fuzziness of information on *p*_12*s*_ described by *μ*_*p*_12__ induces fuzziness of the value of probability measure *P*(*A*_12_) as a discrete fuzzy number *B*_12_. The membership function *μ*_*B*_12__ is defined as

(30) $$\begin{array}{c} \mu_{B_{12}}\left(\;b_{12s}\;\right)=\sup\left(\;\mu_{p_{12s}}\left(\;p_{12s}\;\right)\;\right), \end{array}$$

subject to

(31) $$\begin{array}{c} b_{12s}=\underset{k}{\sum }p_{12s}\left(\;x_{k}\;\right)\mu_{A_{12}}\left(\;x_{k}\;\right). \end{array}$$

As a result, *Z*_12_ = *Z*_1_ + *Z*_2_ is obtained as *Z*_12_ = (*A*_12_, *B*_12_). 

*Scalar Multiplication of Discrete Z-Numbers*. Let us consider a scalar multiplication of a discrete *Z*-number *Z*_*X*_ = (*A*_*X*_, *B*_*X*_): *Z*_*Y*_ = *λ* · *Z*_*X*_, *λ* ∈ *ℛ*. The resulting *Z*_*Y*_ = (*A*_*Y*_, *B*_*Y*_) is found as follows. *A*_*Y*_ = *λA*_*X*_ is determined based on Definition 5.

In order to construct *B*_*Y*_, at first probability distributions *p*_*X*,*l*_, *l* = 1,…, *m*, should be extracted by solving a linear programming problem analogous to 23′-24′. Next, we realize that *p*_*X*,*l*_, *l* = 1,…, *m*, induce probability distributions *p*_*Y*,*l*_, *l* = 1,…, *m*, related to *Z*_*Y*_ as follows:

(32) $$\begin{array}{c} p_{Y}=p_{Y}\left(\;y_{1}\;\right)\setminus y_{1}+p_{Y}\left(\;y_{2}\;\right)\setminus y_{2}+\cdots +p_{Y}\left(\;y_{n}\;\right)\setminus y_{n}, \end{array}$$

such that

(33) yk=λxk,pYyk=pXxk.

The fuzzy set of probability distributions *p*_*X*_ with membership function $\mu_{p_{X}}(p_{X,l})=\mu_{{\tilde{B}}_{X}}\left(\sum_{k=1}^{n}\mu_{{\tilde{A}}_{X}}(x_{k})p_{X,l}(x_{k})\right)$ induces the fuzzy set of probability distributions *p*_*Y*,*l*_ with the membership function defined as

(34) $$\begin{array}{c} \mu_{p_{Y}}\left(\;p_{Y,l}\;\right)=\mu_{p_{X}}\left(\;p_{X,l}\;\right), \end{array}$$

taking into account (32)-(33).

Next, we compute probability measure of *A*_*Y*_, given *p*_*Y*_. Given a fuzzy restriction on *p*_*Y*_ described by *μ*_*p*_*Y*__, we construct a fuzzy number *B*_*Y*_ with the membership function *μ*_*B*_*Y*__:

(35) $$\begin{array}{c} \mu_{B_{Y}}\left(\;b_{Y,l}\;\right)=\sup\left(\;\mu_{p_{Y}}\left(\;p_{Y,l}\;\right)\;\right), \end{array}$$

subject to

(36) $$\begin{array}{c} b_{Y,l}=\underset{k}{\sum }p_{Y,l}\left(\;x_{k}\;\right)\mu_{A_{Y}}\left(\;x_{k}\;\right). \end{array}$$

As a result, *Z*_*Y*_ = *λ* · *Z*_*X*_ is obtained as *Z*_*Y*_ = (*A*_*Y*_, *B*_*Y*_).

Let us now consider the special case of the considered problem of *Z*-rules interpolation, suggested in [50, 51].

Given the *Z*-rules

(37) $$\begin{array}{c} \text{If }X\text{ is }A_{X,1}\text{ then }Y\text{ is }\left(\;A_{Y,1},B\;\right)\\ \text{If }X\text{ is }A_{X,2}\text{ then }Y\text{ is }\left(\;A_{Y,2},B\;\right)\\ \vdots \\ \text{If }X\text{ is }A_{X,n}\text{ then }Y\text{ is }\left(\;A_{Y,n},B\;\right) \end{array}$$

and a current observation

(38) $$\begin{array}{c} X\text{ is }\left(\;A_{X},B_{X}\;\right), \end{array}$$

find the *Z*-value of *Y*.

For this case, as the reliabilities of the *Z*-number based consequents of the considered rules are equal, *B*_*Y*,*k*_ = *B*, according to formula (17) the *Z*-number valued output of the *Z*-rules, *Z*_*Y*_′ = (*A*_*Y*_′, *B*_*Y*_′), is computed as

(39) ZY′∑j=1nwjZY,j=∑j=1nwjAY,j,BY,j=∑j=1nwjAY,j,B,

where *w*_*j*_ = (1/*ρ*_*j*_)/(∑_*k*=1_^*n*^1/*ρ*_*k*_) and *ρ*_*j*_ = ∑_*i*=1_^*m*^ *D*(*Z*_*X*_*i*__′, *Z*_*X*_*i*_,*j*_) = ∑_*i*=1_^*m*^ *D*((*A*_*X*_*i*__′, 1), (*A*_*X*_*i*_,*j*_, 1)) = ∑_*i*=1_^*m*^ *d*(*A*_*X*_*i*__′, *A*_*X*_*i*_,*j*_) as both inputs and the antecedents of the considered *Z*-rules are of a special *Z*-number; that is, they are represented by discrete fuzzy numbers with the reliability equal to 1.

## 5. An Application

Let us consider modeling of a fragment of a relationship between the student motivation, attention, anxiety, and educational achievement [52]. The information on the considered characteristics is naturally imprecise and partially reliable. Indeed, one deals mainly with intangible, nonmeasurable mental indicators. For this reason, the use of *Z*-rules, as rules with *Z*-number valued inputs and outputs based on linguistic terms from a predefined codebook, is adequate way for modeling of this relationship. This rules will help to evaluate a student with given *Z*-number based evaluations of the characteristics. Consider the following *Z*-rules:

 
*The 1st rule*: If motivation is (*M*, *U*), attention is (*H*, *U*), and anxiety is (*L*, *U*), then achievement is (*E*, *U*).
 
*The 2nd rule*: If motivation is (*M*, *U*), attention is (*M*, *U*), and anxiety is (*M*, *U*), then achievement is (*G*, *U*).

**Figure 1:**
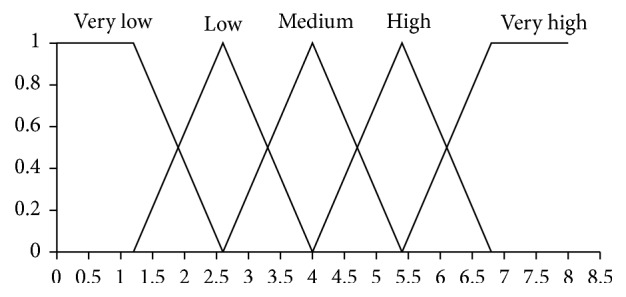
Linguistic terms for a value of motivation.

**Figure 2:**
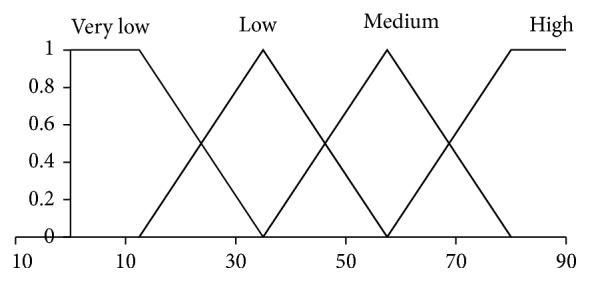
Linguistic terms for a value of attention.

**Figure 3:**
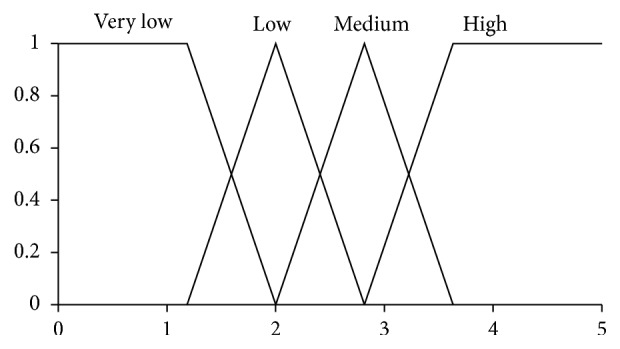
Linguistic terms for a value of anxiety.

**Figure 4:**
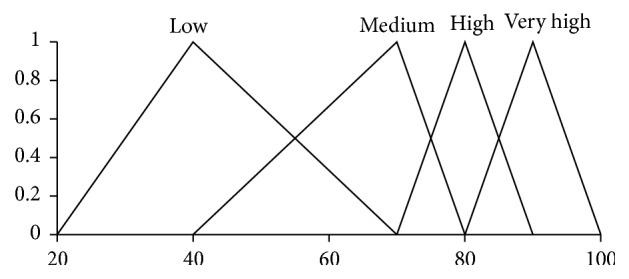
Linguistic terms for a value of achievement.

**Figure 5:**
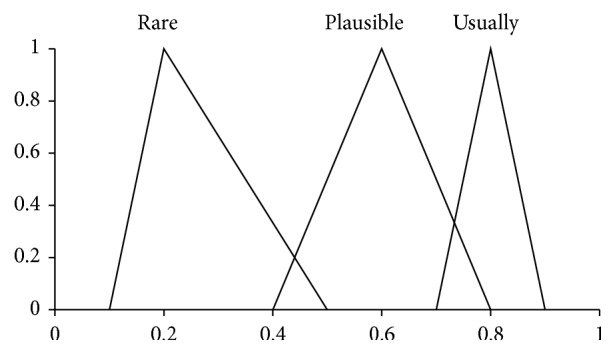
Linguistic terms for reliability of antecedents and consequents.

Here, the pairs (·,·) are *Z*-numbers where uppercase letters denote the following linguistic terms: *H*, High; *L*, Low; *M*, Medium; *G*, Good; *E*, Excellence; *U*, Usually. The codebooks containing linguistic terms of values of antecedents and consequents are given in Figures 1, 2, 3, and 4. The codebook for the degrees of reliability of values of antecedents and consequents is shown in Figure 5.

The considered *Z*-numbers are given below.

The 1st rule inputs:

(40) ZAM=02.6+0.53.3+14+0.54.7+05.4,ZBU=00.7+0.50.75+10.8+0.50.85+00.9;ZAH=057.5+0.568.75+180+190,ZBU=00.7+0.50.75+10.8+0.50.85+00.9;ZAL=01.19+0.51.6+12+0.52.4+02.8,ZBU=00.7+0.50.75+10.8+0.50.85+00.9.

The 1st rule output:

(41) ZAVH=080+0.585+190+0.595+0100,ZBU=00.7+0.50.75+10.8+0.50.85+00.9.

The 2nd rule inputs:

(42) ZAM=02.6+0.53.3+14+0.54.7+05.4,ZBU=00.7+0.50.75+10.8+0.50.85+00.9;ZAM=035+0.546.25+157.5+0.568.75+080,ZBU=00.7+0.50.75+10.8+0.50.85+00.9;ZAM=02+0.52.4+12.8+0.53.2+03.6,ZBU=00.7+0.50.75+10.8+0.50.85+00.9.

The 2nd rule output:

(43) ZAH=070+0.575+180+0.585+090,ZBU=00.7+0.50.75+10.8+0.50.85+00.9.

Consider a problem of reasoning within the given *Z*-rules by using the suggested *Z*-interpolation approach. Let the current input information for motivation, attention, and anxiety be described by the following *Z*-numbers *Z*_1_ = (*Z*_*A*_1__, *Z*_*B*_1__), *Z*_2_ = (*Z*_*A*_2__, *Z*_*B*_2__), and *Z*_3_ = (*Z*_*A*_3__, *Z*_*B*_3__), respectively:

(44) ZA1=02.5+0.53+13.5+0.54+04.5,ZB1=00.6+0.50.65+10.7+0.50.75+00.8;ZA2=025+0.535+145+0.555+065,ZB2=00.6+0.50.65+10.7+0.50.75+00.8;ZA3=01.3+0.52.3+13.3+0.53.65+04,ZB3=00.6+0.50.65+10.7+0.50.75+00.8.

*Z*-interpolation approach based reasoning consists of two main stages.

(1) For each rule compute* dist* as distance *ρ*_*j*_ between the current input *Z*-information *Z*_1_ = (*Z*_*A*_1__, *Z*_*B*_1__), *Z*_2_ = (*Z*_*A*_2__, *Z*_*B*_2__), and *Z*_3_ = (*Z*_*A*_3__, *Z*_*B*_3__) and *Z*-antecedents of *Z*-rules base *Z*_*j*1_ = (*A*_*j*1_, *B*_*j*1_), *Z*_*j*2_ = (*A*_*j*2_, *B*_*j*2_), and *Z*_*j*3_ = (*A*_*j*3_, *B*_*j*3_), *j* = 1,2. For simplicity, we will use the supremum metric *D*(*Z*_*i*_, *Z*_*ji*_) (13):

(45) $$\begin{array}{c} \rho_{j}=\overset{3}{\underset{i=1}{\sum }}D\left(\;Z_{i},Z_{ji}\;\right). \end{array}$$

Consider computation of *ρ*_*j*_ for the 1st and 2nd rules. Thus, we need to determine *ρ*_*j*_ = ∑_*j*=1_^3^ *D*(*Z*_*j*_, *Z*_1*j*_), where values *D*(*Z*_1_, *Z*_11_), *D*(*Z*_2_, *Z*_12_), and *D*(*Z*_3_, *Z*_13_) are computed on the basis of (13). We have obtained the results:

(46) DZ1,Z11dHA1,A11+dHB1,B11=0.9+0.1=1,DZ2,Z1240.1,DZ3,Z131.4.

Thus, the distance for the 1st rule is

(47) $$\begin{array}{c} \rho_{1}=42.5. \end{array}$$

Analogously, we computed the distance for the 2nd rule as

(48) DZ1,Z2,1=1,DZ2,Z2,2=15.1,DZ3,Z2,3=0.8,ρ2=16.9.

(2) Computation of the aggregated output *Z*_*Y*_ for *Z*-rules base by using linear *Z*-interpolation:

(49) ZY=w1ZY,1+w2ZY,2,w1=1/ρ11/ρ1+1/ρ2,  w2=1/ρ21/ρ1+1/ρ2.

The obtained interpolation coefficients are *w*_1_ = 0.28 and *w*_2_ = 0.72. The aggregated output *Z*_*Y*_ is defined as

(50) $$\begin{array}{c} Z_{Y}=0.28Z_{Y_{1}}+0.72Z_{Y_{2}}=\left(\;A_{Y},B_{Y}\;\right). \end{array}$$

We have obtained the following result:

(51) ZAY=072.8+0.578.2+182.6+0.584+089,ZBY=00.68+0.50.73+10.78+0.50.81+00.84.

In accordance with the codebooks shown in Figures 4 and 5, we have achievement is “*High*” with the reliability being “*Usually*.” This linguistic approximation is made by using similarity measure between the obtained output and fuzzy sets in the codebooks.

## 6. Conclusion

A concept of a *Z*-number suggested by Zadeh is a key to computation with imprecise and partial reliable information. In this paper, we propose applying distance of *Z*-numbers to approximate reasoning within IF-THEN rules with *Z*-numbers-based antecedents and consequents.

A real-world application of the suggested research has been provided to illustrate its validity and potential applicability.
